# An Updated View of the Importance of Vesicular Trafficking and Transport and Their Role in Immune-Mediated Diseases: Potential Therapeutic Interventions

**DOI:** 10.3390/membranes12060552

**Published:** 2022-05-25

**Authors:** Miguel A. Ortega, Oscar Fraile-Martinez, Cielo Garcia-Montero, Miguel Angel Alvarez-Mon, Ana Maria Gomez-Lahoz, Agustin Albillos, Guillermo Lahera, Javier Quintero, Jorge Monserrat, Luis G. Guijarro, Melchor Alvarez-Mon

**Affiliations:** 1 Department of Medicine and Medical Specialities, Faculty of Medicine and Health Sciences, University of Alcala, 28801 Alcala de Henares, Spain; oscarfra.7@hotmail.com (O.F.-M.); cielo.gmontero@gmail.com (C.G.-M.); maalvarezdemon@icloud.com (M.A.A.-M.); alahoz1199@gmail.com (A.M.G.-L.); agustin.albillos@uah.es (A.A.); guillermo.lahera@gmail.com (G.L.); jorge.monserrat@uah.es (J.M.); mademons@gmail.com (M.A.-M.); 2 Ramón y Cajal Institute of Sanitary Research (IRYCIS), 28034 Madrid, Spain; luis.gonzalez@uah.es; 3 Cancer Registry and Pathology Department, Principe de Asturias University Hospital, 28806 Alcala de Henares, Spain; 4 Department of Psychiatry and Mental Health, Hospital Universitario Infanta Leonor, 28031 Madrid, Spain; 5 Unit of Biochemistry and Molecular Biology, Department of System Biology (CIBEREHD), University of Alcala, 28801 Alcala de Henares, Spain; 6 Department of Legal Medicine and Psychiatry, Complutense University, 28040 Madrid, Spain; fjquinterog@yahoo.es; 7 Psychiatry Service, Center for Biomedical Research in the Mental Health Network, University Hospital Príncipe de Asturias, 28806 Alcala de Henares, Spain; 8 Immune System Diseases-Rheumatology, Oncology Service an Internal Medicine (CIBEREHD), University Hospital Príncipe de Asturias, 28806 Alcala de Henares, Spain

**Keywords:** exosomes, cellular traffic, extracellular vesicles, immune-mediated diseases, therapeutic applications

## Abstract

Cellular trafficking is the set of processes of distributing different macromolecules by the cell. This process is highly regulated in cells, involving a system of organelles (endomembranous system), among which are a great variety of vesicles that can be secreted from the cell, giving rise to different types of extracellular vesicles (EVs) that can be captured by other cells to modulate their function. The cells of the immune system are especially sensitive to this cellular traffic, producing and releasing different classes of EVs, especially in disease states. There is growing interest in this field due to the therapeutic and translational possibilities it offers. Different ways of taking advantage of the understanding of cell trafficking and EVs are being investigated, and their use as biomarkers or therapeutic targets is being investigated. The objective of this review is to collect the latest results and knowledge in this area with a specific focus on immune-mediated diseases. Although some promising results have been obtained, further knowledge is still needed, at both the basic and translational levels, to understand and modulate cellular traffic and EVs for better clinical management of these patients.

## 1. Introduction

Cellular trafficking is the process by which proteins and the rest of macromolecules are distributed throughout the cell and released to the extracellular space (exocytosis) or internalized (endocytosis) through the so-called endomembrane system [1]. This system is mainly composed of a series of membranous organelles, among which the rough endoplasmic reticulum, smooth endoplasmic reticulum, Golgi apparatus (GA), lysosomes, and different types of vesicles stand out. The endomembranous system is fundamental for the interaction of the cell with the environment, playing a key role in the development of various homeostatic and adaptive mechanisms [2]. A growing number of studies seem to indicate the relevance of these communication and transport systems in the development of various pathologies [3,4]. Among the main components transported in the endomembrane system, proteins and some oligonucleotides stand out for their important signalling and modulating functions [5,6]. Other macromolecules, such as lipids and complex carbohydrates, can also be transported [7,8]. Extracellular vesicles (EVs) are central elements of cellular trafficking that contain many of these components, having attracted growing interest because of their biological functions [9]. There are different types of EVs, the best characterized being exosomes, microvesicles, and apoptotic bodies, differing from each other by their size, content, biogenesis, forms of release, and function [10].

In this sense, cellular trafficking and EVs appear to have a great impact on the immune system (IS), having a close influence on its function [11]. On the one hand, the IS receives and integrates signals from different cells of the organism, as well as from pathogens through this endomembrane system, modulating various cellular signalling pathways [12]. In turn, cellular trafficking and EVs are crucial for the subsequent immune response, and an alteration of cell traffic can lead to the appearance of various pathologies of the immune system, including autoimmune diseases or immunodeficiencies [13,14,15]. Because of that, a growing body of evidence supports the relevance of targeting and influencing cell trafficking and EVs in a wide variety of pathologies in which the IS is involved, including the so-called immune-mediated diseases (IMIDS) [16,17,18].

The present review aims to analyse, in a general way, the different biological mechanisms involved in cell traffic and transport to describe the importance of the endomembrane system in different cellular processes. The processes underlying defective cell trafficking and its relationship with IMIDs will be reviewed to help find therapeutic targets that can be applied in translational medicine for these pathologies.

## 2. Biological Basis of Cell Trafficking

### 2.1. General Mechanisms of Cellular Transport and Traffic

The cell is a highly dynamic organism that receives, integrates, and sends signals to its environment. As mentioned above, this function falls directly on the endomembranous system, composed of several organelles of the cell that work in a coordinated manner to favour the adaptation of the cell to the environment. The endoplasmic reticulum (ER) is a network of dynamic sheets and tubules that project from the nuclear envelope and play a great variety of roles in cells, participating in the synthesis, folding, and transport of proteins (rough ER) and in the synthesis and metabolism of lipids and carbohydrates, as well as intracellular calcium storage (smooth ER) [19,20]. The GA is a highly important organelle that is closely linked to the ER, acting as a post-translational modification factory and a centre of protein and lipid trafficking in the cell. It appears to be composed of five to eight flattened cisterns that can be subclassified into three large groups: a cis network oriented towards the ER that receives components from it; cis, medial, and trans cisternae with protein and lipid processing functions that also have glycosylation enzymes; and a trans network oriented towards the plasma membrane that classifies and sends cargo molecules to their destination [21,22]. This trafficking of molecules between the ER, the GA, and the appropriate cell fate is performed by the vesicles, which frequently go to the plasma membrane to exit the cell [23]. This ER–Golgi traffic toward the plasma membrane is known as exocytosis, and involves secretion into the medium or the membrane of the transported vesicles.

Exocytosis can be constitutive or regulated. While the first occurs in all cells, the second occurs in specialized cells such as neurons and exocrine or endocrine cells [24]. The fusions of the vesicles with the membrane can be direct (complete fusion), in the form of ‘kiss-and-run’ mechanisms, where the vesicle is fused in an incomplete manner, forming a pore that communicates with the cell exterior, or it can be in the form of compound fusion, where the vesicle fuses with others before or just after joining the plasma membrane and being released to the exterior [25,26]. A large proportion of the exocytosis products are returned to the cell from the opposite process, endocytosis, which is temporally and spatially coordinated with exocytosis [27]. There are different types of endocytosis, which can be classified into phagocytosis and pinocytosis. The latter can be broken down into macro- and micropinocytosis, depending on the size of the internalized particle, and it may be mediated by classical pathways (caveolae and clathrin) or by pathways independent of these components [28].

The endocytosed vesicle, in addition to being composed of substances from the external environment, incorporates some of the plasma membrane, with its constituent lipids, receptors, and proteins, leading to the formation of the early endosome [29]. The early endosome serves as an initial classification link that will determine the route that the endocytosis component follows, which can be mainly three different pathways: (1) it is directed back to the plasma membrane (recycling); (2) it goes to the trans Golgi network, where by retrograde transport the product is mobilized back to the ER; or (3) the late endosome is formed [30]. Late endosomes (also known as multivesicular bodies, MVBs) form after the maturation of the early endosome and represent a central axis for the incoming traffic from the endocytosis or biosynthetic pathways (from the GA). They also regulate the trafficking of their internalized products towards the plasma membrane (recycling), the GA (retrograde transport), or lysosomes, which are responsible for the degradation of the vesicles contained within [31,32].

**Figure 1:**
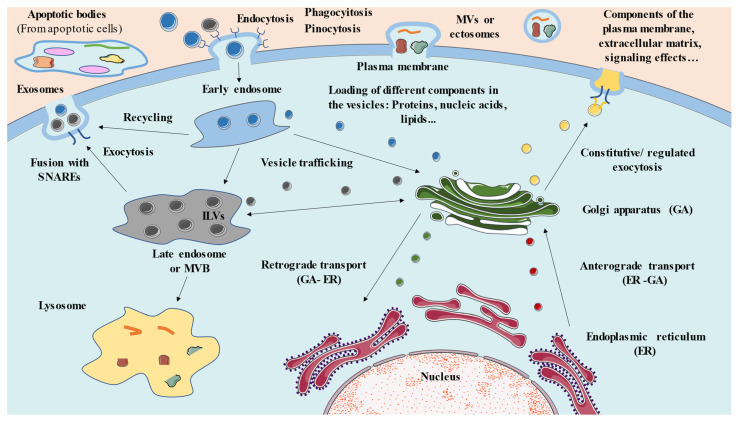
Summary of the main parties involved in cellular and vesicular traffic. There is communication between the ER and the GA, where the vesicles are packed and classified, transporting different contents in both directions (anterograde and retrograde transport). Some of these vesicles are sent to the plasma membrane to be secreted by a process of constitutive or regulated exocytosis (mainly mediated by the SNARE complex). By endocytosis, different vesicles and substances from the medium are captured, entering the cell and forming the early endosome. The early endosome can recycle these components back to the cell exterior, send them to the GA, or give rise to a secondary endosome, also called multivesicular bodies (MVBs), as this structure contains multiple smaller vesicles known as intraluminal vesicles (ILVs). There is important communication between the GA and MVBs, yielding a bidirectional exchange of vesicles that can be secreted, giving rise to exosomes or becoming part of lysosomes for their subsequent degradation. Likewise, other types of extracellular vesicles, ectosomes or microvesicles (MVs), can be formed directly from the plasma membrane. It is noteworthy that each vesicle can have very diverse contents, including different types of proteins, lipids, and nucleic acids.

Endosomes are also fundamental in the process of transcytosis, a type of cellular trafficking that occurs in polarized cells in which molecules that enter by endocytosis are transported from one end of the cell to the other, where they are released by a process of exocytosis [33]. Figure 1 summarizes the main mechanisms presented in this section, as well as the different components involved.

### 2.2. Relevance of Extracellular Vesicles in Cell Trafficking

Just as it is important to know the mechanisms involved in cell transport, it is interesting to delve into what products the cells can transport and what type of strategies they use to do so. As indicated above, the cellular products can be synthesized and packaged by the ER and GA and transported in the form of vesicles to different cellular locations, such as the membrane or late endosomes, whence they will be directed to the appropriate destination. A great variety of markers set the destiny of the vesicles [34,35,36]. EVs are a central part of the cellular trafficking and involve the transportation and release of several critical molecules from the cell to the extracellular matrix. Due to the complexity of EVs and the scope of this review, we focus only on the most representative ones: exosomes, microvesicles, and apoptotic bodies. The origin of these EVs that are subsequently discussed are summarized in Figure 1. In this section, we review the most relevant characteristics of these three types of EVs, focussing on their formation, content, and biological roles.

#### 2.2.1. Exosomes

Exosomes are the smallest EVs (between 30 and 100 nm across) and are released by all cells of the organism, including pathogenic microorganisms or the microbiota, fulfilling important functions in intercellular communication [37]. This communication not only occurs between neighbouring cells, but exosomes have been detected in practically all human fluids, showing the role of these components at the systemic level [38]. In fact, exosomes carry a key ‘molecular fingerprint’ of the donor cell by being carriers of a great variety of molecules, including lipids, proteins, glycoconjugates, and nucleic acids such as DNA and different types of RNA [39].

Exosomes are formed in late endosomes or MVBs (Figure 1), and more specifically within the intraluminal vesicles (ILVs) that are generated inside. During the process of secondary endosome formation, certain proteins are incorporated into the invaginating membrane, while other cytosolic components are directly absorbed and enclosed within the ILVs [40]. The internalized molecules can be recycled after their endocytosis or can be loaded in the ILVs through a great variety of mechanisms, among which are endosomal classification complexes required for transport (ESCRT), formed by four ESCRT proteins (0–III) that function in a coordinated manner to facilitate the formation of MVBs and vesicles and the classification of their protein loads [41,42]. The importance of other components in the biogenesis of exosomes has also been highlighted, including some lipids, such as ceramides and cholesterol; different proteins, such as Alix; tumour susceptibility gene 101 (Tsg101); flotillin 1; arrestins such as Arrestin domain containing 1 (Arrdc1); some members of the tetraspanin family (such as CD9, CD63, and CD81); and different GTPases, such as ADP ribosylation Factor 6 (ARF6) and members of the Rab family [43,44,45,46]. Overall, experts identify three major pathways of exosome biogenesis. The first one is the ESCRT-mediated biosynthesis, by which ESCRT-0 and -I recruit cargos and ESCRT-II, which then recruits ESCRT-III and in turn, ESCRT-III promotes ILV budding. Then, the vacuolar protein sorting 4A (Vps4) complex ensures final membrane scission and/or ESCRT recycling [47,48]. It is of note that cargo transfer into ILVs is highly selective. Two models have arisen to explain this process: In one model, the early acting ESCRT machinery forms a subdomain adjacent to ESCRT-III filaments, which spiral inwards toward a preferred radius of curvature. In this model, the cargoes are transferred between the early- and late-acting ESCRT complexes. In an alternative model, a subdomain formed by the early-acting ESCRT machinery nucleates ESCRT-III filaments that spiral outward to surround cargoes destined for incorporation into ILVs. ESCRT-0, ESCRT-I, and ESCRT-II must dissociate from the endosomal membrane to avoid engulfment into ILVs [49]. Likewise, the protein ALIX appears to play a key role on the exosome biogenesis, being able to interact with specific proteins of the ESCRT complex such as Tsg101—a subunit of ESCRT-I—and with syntenin, the adaptor protein of the proteoglycan syndecan [50]. Interestingly, this effect appears to be mediated by ARF6 and phospholipase D2 (PLD2). These components appear to control syntenin-ALIX ILV formation and thereby subsequent exosomal release with effects on ESCRTs that are selective or depend on context (that is, specific cargo) [45]. (1) Tetraspanin-mediated biogenesis appears to be important in the recycling pathways between plasma membrane and cellular organelles and regulating biosynthetic maturation and trafficking [51]. In this sense, previous studies have described the relevance of the tetraspanins in the EV release of specific components in physiological and pathological conditions, and how their targeting is associated with an abnormal cargo in the exosomes, despite further studies being needed to elucidate the molecular mechanisms [52]. In addition, there are other tetraspanins, such as CD6 negatively regulating exosome biogenesis, directing MVB cargoes for lysosomal degradation [53]. (2) Lipid-mediated biogenesis has a central role of ceramide and cholesterol lipid domain recruit factors, including flotillins and the autophagy-related protein LC3. In turn, some of these factors recruit certain members of the Rab family, interacting with these factors. For instance, flotillin recruits Rab31, which is involved in EGFR cargo in MVB. Rab27a/b regulates fusion of MVBs at the plasma membrane for exosome release and Rab7 induces MVB fusion with lysosomes, although it is directly regulated by Rab31 [54]. For a detailed view of the biogenesis of exosomes, see the chapter written by Kang et al. [55].

As mentioned above, these secondary endosomes can either fuse with lysosomes and be degraded or be directed to the membrane. Fusion of the MVB with the cell membrane will release the exosomes to the outside of the cell. For this fusion to occur, many energy barriers must be overcome, although the specific molecular mechanism involved in the fusion of MVBs with the plasma membrane has not been fully characterized. There are a great variety of protein–lipid and protein–protein interactions in which the importance of some proteins is known, such as soluble *N*-ethylmaleimide-sensitive factor attachment protein receptors (SNAREs) [56]. SNARE proteins are anchored to the membranes, playing a critical role on membrane fussion. The SNARE hypothesis suggests that SNAREs are contained in the vesicles (v-SNAREs) and interact with t-SNAREs (on the target compartment) to form a trans-SNARE complex [57]. After fusion, the trans-SNARE complex becomes a cis-SNARE complex on the target membrane, which then binds to soluble NSF-attachment protein (α-SNAP) and N-ethylmaleimide-sensitive fusion protein (NSF) to form a transient 20S complex. Afterwards, the ATP is hydrolysed by NSF, resulting in the disassembly of the SNARE complex to allow the recycling of v-SNARE and t-SNARE to be used for the next round of membrane fusion [58]. This process is highly regulated by a long list of tethering factors and Sec1/Munc18 (SM) proteins, which have physical interactions with SNAREs [59]. In the MVBs, there are some important tethering factors such as homotypic fusion and vacuole protein sorting (HOPS), interacting with the SNAREs contained in this structure and influencing their fate [58]. Different members of the Rab family are also crucial for vesicular trafficking among different organelles, also mediating membrane fussion and exosome release [56,60].

Exosome contents also represent an important point of study in this field. Given the great variety of proteins, lipids, and nucleic acids that can be present in exosomes, the ExoCarta database was created to collect all the components identified in studies of exosomes as well as the interaction of their components and prominent biological effects [61,62]. More than 7540 RNAs, 1116 lipids, and 41,860 proteins can be transported in exosomes, which means that at least 25% of the human proteome can be secreted via exosomes [63]. Exosomes are covered by a lipid bilayer with abundant amounts of cholesterol, sphingomyelin, glycosphingolipids, and phospholipids, with a high predominance of monounsaturated fatty acids in their composition [64,65]. This specific composition of exosomes makes them more rigid than the plasma membrane, which can also contribute to a greater resistance to degradation, increasing their efficiency as messengers of different biomolecules [9]. These lipids are not only part of the structure of exosomes but also help their formation and release into the environment. DNA molecules and different types of RNA have been detected within exosomes. In the case of DNA, exosomes can be important carriers of genetic and mutagenic information from tumour cells, which can favour the progression of different types of cancer [66]. Exosomes can contain genomic DNA of all chromosomes, although their size is usually less than 10 kilobases of DNA [67]. Exosomes can also be carriers of mitochondrial DNA under both physiological and pathological conditions [68,69,70]. As for RNA (EV-RNA), a wide variety of subtypes have been detected in exosomes, especially microRNAs (miRNAs), transfer RNA (tRNA), and messenger RNA (mRNA), the first two making up approximately 15% and the last approximately 33% of EV-RNA [71]. The other 50% is repeated RNA regions, although other types of noncoding RNAs can also be observed, such as long noncoding RNAs (lncRNAs) or small interfering RNAs (siRNAs) [72,73]. The evidence seems to support the functionality of EV-RNA in receptor cells, acting not only in a paracrine way but also in a systemic way, influencing the transcriptional regulation of a great variety of proteins [9]. As mentioned above, there are plenty of proteins contained in the exosomes, including a wide variety of receptors, transcription factors, and enzymes, which can lead to notable phenotypic changes in receptor cells.

Finally, exosomes are captured by other cells after recognition by the target cell receptors or by direct fusion with the plasma membrane, releasing their content directly into the cell. They can also be internalized by phagocytosis, macropinocytosis, or different types of micropinocytosis, giving rise to early endosomes. Then, inside the recipient cell, these exosomes can either be recycled back to the plasma membrane and released or be directed to MVBs, where they will fuse with lysosomes for degradation or their content will be released in the cytosol, nucleus, or ER, where they will exercise their cellular functions [53]. Finally, exosomes are important in a wide variety of biological processes, suggesting their potential use as biomarkers and therapeutic agents/targets [74,75,76].

#### 2.2.2. Microvesicles

Microvesicles (MVs), also known as ectosomes, are EVs between approximately 100 and 1000 nm across. In a similar way to exosomes, they play a key role in intercellular communication, transporting bioactive molecules from the donor cell to the recipient [77]. Unlike exosomes, MVs originate directly by sprouting from the plasma membrane and are detected in all cells, tissues, and body fluids of the organism [78].

The biogenesis and content of MVs have some similarities and differences with exosomes. On the one hand, they arise directly from the plasma membrane, as a result of coordinated work between membrane phospholipids and the contractile action of the cellular cytoskeleton [79]. Throughout this process of biogenesis, the importance of ATP-binding cassette transporter 1 (ABCA1) in the induction of asymmetry and imbalance of the plasma membrane is recognized. Simultaneously, other components also involved in the formation of exosomes, such as ARF6 and PLD2, are also involved in the biogenesis of MVs, initiating a cellular signalling cascade that results in the contraction of actin and myosin and the release of MVs [55]. In more detail, ARF6 as well as ARF1 induce the biosynthesis of microvesicles via activation of RhoA, which induces the phosphorylation of the myosin light chain (MLC) via a Rho-associated protein kinase (ROCK) signalling pathway [80]. Diaphanous Related Formin 3 (DIAPH3), glutaminase, hyaluranon synthase, and protein–protein crowding are also involved in the biogenesis of microvesicles, although more studies are required to elicit the precise molecular mechanism by which they work [81]. In addition, some members that are involved in the biogenesis of exosomes also appear to be implicated in the biogenesis of MVs such as ESCRT, ALIX, TSG-101, and ARRDC1, among others [82,83]. However, the role of these components may be slightly different in the biogenesis of MVs. For instance, ARRDC1 and other arrestins interact with HECT ubiquitin ligases (WWP1, WWP2, and Itch), ALIX, and Tsg101, thereby influencing ESCRT activation [82]. Likewise, the inhibition of Vps4 disturbs the production and release of MVs [84]. Furthermore, Rab22a can also be involved in the biogenesis of MVs under hypoxic conditions according to previous studies [85]. Despite the biogenesis of MVs and exosomes being frequently coupled, it seems that activated cells present a greater release of MVs in a mechanism dependent on intracellular calcium, which modulates and regulates the aforementioned components [86]. Lipids are also crucial for the synthesis of MVs. The modification of the plasma membrane asymmetry can be implicated in the production of MVs by several mechanisms including (1) through the action of aminophospholipid translocases, (2) the translocation on the outer plasma membrane of the acid sphingomyelinase (aSMase) promoting the budding of microvesicles, and (3) by modification of the lateral pressure of phospholipids via the phosphatidylserine (PS)-binding protein on the inner leaflet or sphingomyelin/cholesterol-binding protein on the outer leaflet [87].

Then, just like exosomes, MVs may interact with cellular receptors, fuse with the membrane, and release their contents directly into the cytosol or be internalized by different mechanisms, forming early endosomes and then being recycled or becoming part of an MVB [88].

With respect to their contents, like exosomes, MVs carry different lipids and proteins of the plasma membrane, as well as other proteins and nucleic acids contained nearby in the cytoplasm, which lead to phenotypic changes in receptor cells [89]. MVs were found to be selectively enriched in cytoskeletal elements, cytoskeleton-associated proteins (e.g., actin, actinin, dynamin, myosins, tubulin, and VDAC1/2), and septins (along with their associated binding partners) [90]. Integrins, selectins, and CD40 are frequently recognized in MVs, and as tetraspanins can also be detected on cell membranes, they may take part in MVs as well, especially CD9 [51]. In general, MVs are carriers of different proteins involved in the remodelling of the extracellular matrix, with important functions in both physiological and pathological conditions [91]. In addition, they seem to have a central role in endothelial function, coagulation, or inflammatory response, as well as in the development of different cardiometabolic disorders [77]. Thus, the use of MVs as diagnostic, prognostic, and therapeutic tools has also been evaluated in different studies [92,93].

#### 2.2.3. Apoptotic Bodies

Apoptotic bodies (ApoBDs) are large EVs, generally between 1 and 5 microns across, although recent studies have shown that there are ApoBDs larger than 5 µm and smaller than 1 µm [94]. Along with exosomes and MVs, ApoBDs are key information messengers from donor to recipient cells, but they are not produced in MVBs nor in the plasma membrane. Their origin is linked to programmed cell death or apoptosis. The process of apoptosis develops in several phases, concluding with the disintegration and packaging of the cellular content in different vesicles from the membrane, giving rise to ApoBDs. Further, three steps are recognized in the formation of ApoBDs: plasma membrane blebbing (step 1), the formation of thin apoptotic membrane protrusions (step 2), and finally, the fragmentation into individual ApoBDs (step 3) [95]. The first step involves the formation of large circular bulges (known as blebs) on the surface of the apoptotic cell due to an actomyosin contraction which leads to an enhanced hydrostatic pressure and bleb formation [96]. In this sense, previous studies have given a central role to the ROCK1 signalling, despite other molecules possibly also being implicated in this process [97]. The second step involves the generation of long apoptotic membrane protrusions known as apoptopodia and beaded apoptopodia. These structures are key for the later formation of ApoBDs, although they work in a different manner. Whereas the apoptopodia can drive the formation of ApoBDs by separating membrane blebs, a single beaded apoptodia can form 10–20 individual ApoBDs that are generally uniform in size [98,99]. Among the main molecular regulators on this step, Pannexin 1 (PANX1) channels, Plexin B2 (PlexB2) receptors, the cytoskeletal network, and vesicle trafficking should be highlighted. The first, PANX1, is a negative regulator of apoptopodia and beaded apoptopodia formation, whereas PlexB2 and vesicle trafficking positively regulate their formation [100]. Notwithstanding further research being required, the release of ApoBDs may be facilitated by shear force, an abscission-like process, or through interactions with neighbouring phagocytes [101]. In addition, the activation of some of the machinery involved in the formation of exosomes and MVs such as the ESCRT complex and related proteins (Tsg-101, ALIX) appears to negatively regulate apoptosis and ApoBDs’ formation [102]. Overall, the main proteins involved in the biogenesis of ApoBDs, MVs, and exosomes are collected in Table 1.

The contents of ApoBDs can be varied, including micronuclei, portions of cytoplasm, degraded proteins, chromatin remnants, DNA fragments, and even intact organelles. Unlike other EVs, ApoBDs contain more RNA, as well as proteins, lipids, and nucleic acids of greater size and length [103].

ApoBDs and cells are phagocytosed by different cell types, especially by macrophages, in a process called spherocytosis. Apoptotic cells display ‘find me’ and ‘eat me’ signals that are recognized by phagocytic cells. The most characteristic example is PS, a phospholipid that normally is found inside the membrane but translocates to the outside of the membrane after entering apoptosis, acting as a signal for its phagocytosis [104]. Importantly, this PS also appears in ApoBDs, differentiating them from exosomes and MVs [98].

Although exosomes and MVs have been studied in greater depth, it is thought that ApoBDs play a central role in the regulation of the process of apoptosis and modulation of the IS [105]. In fact, ApoBDs have a strong influence on phagocytic cells, regulating their metabolism and inhibiting inflammation, also thanks to the products contained within them. Understanding the role of ApoBDs in receptor cells is equally fundamental to unravelling the onset of different diseases, opening potential therapeutic lines for their treatment [106].

## 3. Cell Trafficking in Immune-Mediated Diseases: Translational Approach

### 3.1. Importance of Cell Trafficking and EVs in Immune-Mediated Inflammatory Diseases

Cellular traffic mediated by the endomembrane system is a key element of the functioning of the IS that, in turn, integrates and responds continuously and appropriately to the EVs it receives. Failures in cellular traffic or in the reception of EVs and signals from the environment lead to a wide variety of pathologies related to IS [12,107]. IMIDs are a group of IS disorders characterized by an elevated inflammatory response sustained over time [108]. IMIDs are multifactorial diseases that are caused by the interaction of a set of genetic and environmental mechanisms, and they bring an important socioeconomic burden [109,110]. IMIDs include a wide variety of pathologies, such as rheumatoid arthritis, inflammatory bowel disease, spondyloarthritis, psoriasis, atopic dermatitis, connective tissue disorders, asthma, and autoimmune neurological diseases such as multiple sclerosis [111].

IS cells are very active cells, especially during IMID. This implies that cell trafficking and the endomembrane system will have a central role in these pathologies. On the one hand, the importance of the ER as a link between the IS and cellular metabolism is widely accepted, and failures in the function of the ER lead to a wide variety of immune and inflammatory pathologies [112]. For example, ER stress (which is associated with an abnormal functioning of this organelle and the accumulation of misfolded proteins and alteration of cellular traffic, among other effects) leads to a release of different proinflammatory cytokines, including interleukin 1-β (IL-1β) and IL-6 [113,114]. On the other hand, the GA is also a critical organelle that regulates not only cellular trafficking but also the activation of the innate IS, including the activation of the NLRP3 inflammasome, the production of type I interferon, and Toll-like receptor (TLR-3) and retinoic-acid-inducible gene receptor (RLR) signalling [115]. Patients with some autoimmune diseases have elevated autoantibodies against the GA, which supports the importance of this organelle in immunopathological conditions [116]. Similarly, under inflammatory conditions, the processes of exocytosis and endocytosis are especially active. There is a marked increase in regulated exocytosis in the different inflammatory cells through the so-called secretion granules [117].

Likewise, IS cells will capture a wide variety of pathogen-associated molecular patterns (PAMPs) or damage-associated molecular patterns (DAMPs), carrying out different types of endocytosis and integrating the signals from the medium [118]. In this sense, the importance of endosomes and lysosomes in the IS clearly established, regulating not only the degradation or recycling of external materials but also participating in the presentation of antigens or in the activation of cellular receptors such as TLRs [119].

On the other hand, EVs have very important functions in the regulation of the immune response. As mentioned above, all cells release exosomes and MVs, including tumour cells, as well as the different microorganisms in the body (microbiota) and the different infectious agents [120,121,122]. IS cells release but above all capture different EVs from the environment, thus regulating the immune response developed. EVs have important effects on innate and adaptive IS cells, participating both in the presentation of antigens and activation of the IS as well as in its modulation and suppression [123]. For example, placental cells are important in the tolerization of the IS during foetal development thanks to the secretion of EVs containing human leukocyte antigen G (HLA-G) [124]. In turn, in vitro studies have shown how immune cells tolerated by this mechanism can do the same, thus facilitating the tolerization of all cells [125]. Similar mechanisms are used by tumour cells to evade the IS and promote inflammation, which favours their progression and development [126].

In the case of IMIDs, different mechanisms of interest have been studied in which these EVs can play a central role. For example, in patients with rheumatoid arthritis there is an increase in the autoantigens transported in EVs as well as in the transport of tumour necrosis factor α in their membranes, which leads to a permanent activation and resistance to T-cell apoptosis, with pathophysiological mechanisms of great relevance to this disease [127].

EVs carry multiple enzymes that contribute to the degradation of the extracellular matrix of cartilage or of different cytokines and microRNAs that promote inflammation in the joints [128,129]. In patients with inflammatory bowel disease, there are high levels of EVs, as well as a wide variety of components both in their membrane and in their interior, involved in the immunomodulation and alteration of the intestinal barrier [130,131]. Similar mechanisms have been described in other autoimmune diseases, such as systemic lupus erythematosus, type 1 diabetes, psoriasis, vitiligo, or preeclampsia, as well as in respiratory or neuroinflammatory disorders, which supports the importance of these EVs in pathological activation. of the IS [17,107]. As a whole, the accumulated evidence suggests the enormous potential of numerous therapeutic strategies based on intra- and intercellular vesicular traffic and transport as a treatment for IMIDs, which is reviewed below.

### 3.2. Therapeutic Strategies Aimed at Cell Trafficking

**Figure 2:**
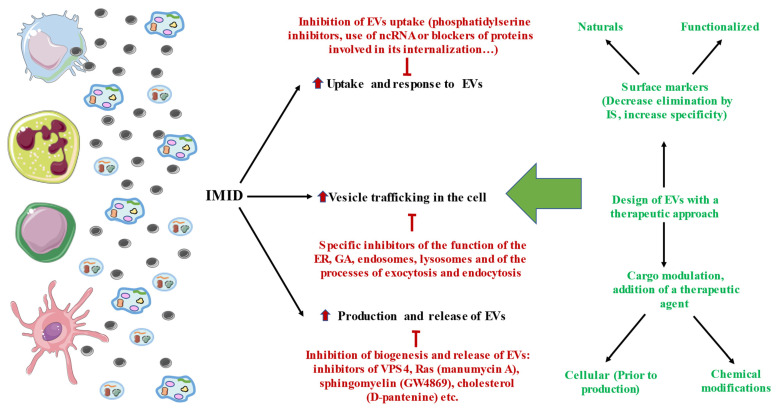
Overall summary of the therapeutic strategies currently pursued to limit the influence of high cellular traffic, production, release, and uptake in the IS. There are different approaches aimed at cell trafficking and its organelles, as well as at EVs, with some promising but still preclinical results. On the other hand, researchers are also beginning to design EVs that can be used for therapeutic purposes. In these cases, it is important to try to ensure their specificity and avoid their elimination by cells of the IS, so the selection of their membrane components should be oriented to this end. In the same way, modulating their contents and even adding a specific therapeutic agent could bring multiple benefits to the clinical management of IMIDs. For this, different approaches are being evaluated, such as modifying their content before their production (at the cellular level) or after being released by the cells, through chemical modifications.

Due to the important activity of the IS in IMIDs, a wide variety of strategies aimed at vesicular traffic as a translational approach to these pathologies are currently being investigated. On the one hand, and as indicated above, the vesicles transported in the different fluids of the human body can be used as important diagnostic, prognostic, or predictive biomarkers. The therapeutic approach is more complex, and two major approaches are currently being studied: (1) targeted therapy against EVs and vesicular traffic, and (2) the use of EVs and their contents as a therapeutic alternative or the use of EVs as nanocarriers carrying specific therapeutic agents (Figure 2). In this section, we summarize the main approaches used based on vesicular traffic in IMIDs.

#### 3.2.1. Therapy Directed at Vesicular Traffic

Various therapeutic strategies are being developed that target the biosynthesis of different vesicles, their trafficking, and their release to the extracellular medium, as well as treatments directed at specific cellular organelles. In particular, the efficacy of the inhibition of exocytosis and endocytosis in infectious processes or cancer has been postulated [132]. Despite some promising preclinical results, there are certain limitations to this approach, including the selective permeability of biomembranes, efflux transporters, and lysosomes [133].

Therapy directed to cell trafficking is seen as a potential approach to IMIDs. To date, there are no therapeutic approaches specifically directed to ER as a strategy to influence cell trafficking. However, notwithstanding the evidence for these components in in vivo studies is still very limited, there are certain therapeutic agents targeting ER stress in immune cells, such as IRE1α, XBP1, PERK, ATF6, and CHOP, indirectly affecting cell trafficking [134]. There are also GA inhibitors, although most studies have focussed on genetic diseases, cancer, and infectious diseases [135]. Patra et al. [136] described how the use of a specific inhibitor of endosomal TLRs (TAC5 and its derivatives) attenuated the inflammatory response in some IMIDs, such as psoriasis. Finally, there is a wide arsenal directed against different components of lysosomes. In a recent review written by Bonam et al. [137], the authors discuss the main inhibitors of lysosomes in different autoimmune and inflammatory diseases, warning of the importance of developing specific therapeutic agents aimed not at all lysosomes but at the components that are altered in a specific way in a specific pathology.

On the other hand, different components have been developed that specifically target EVs—their biogenesis, loading, release, and uptake by recipient cells. On this topic, there have been some interesting results with respect to the inhibition of Vps4, which acts in a coordinated manner with ESCRT in the biogenesis of EVs [48]. The inhibition of this component results in a reduction in exosomes and MVs [114]. In the field of immunology, the inhibition of Vps4 leads to a decrease in EVs in T cells [138]. Other therapeutic targets include proteins of the Ras family (manumycin A), sphingomyelinases (GW4869, imipramine), and cholesterol synthesis inhibitors (d-panthenine) [139].

Likewise, it is important to study the effects of the inhibition of these components. For example, the inhibition of sphingomyelinase by GW4869, despite leading to a lower release of exosomes, induces a greater release of MVs, so the possible consequences in each pathology should be studied [140]. Gon et al. [141] demonstrated that the treatment of mice with GW4869 significantly improved airway inflammation after exposure to allergens due to the lower release of exosomes that recruited and activated eosinophils. The use of indomethacin, an inhibitor of the ABCA3 pump, increased the efficacy of some antitumour treatments by inhibiting the release of the antitumor agents from exosomes [139].

Another potential therapeutic strategy is to avoid endocytosis and uptake of EVs. There are different ways to achieve this, including the inhibition of phosphatidyl serine in the extracellular membrane, inhibition by noncoding RNAs of proteins involved in capturing EVs, and the blocking of proteins involved in the internalization of extracellular components [130]. In IS cells, integrins and immunoglobulins have important roles in the interaction with EVs, and in fact the use of specific antibodies against the binding sites of CD11a or its ligand, the glycoprotein ICAM-1 (Intercellular Adhesion Molecule 1), seems to reduce uptake of EVs in dendritic cells [142]. Similar results have been obtained by the inhibition of other proteins in different IS cells, showing the importance of protein–protein interactions in the uptake of EVs in immune cells [68]. The opposite happens in the case of ApoBDs, as spherocytosis is ineffective in a wide variety of inflammatory pathologies. In these diseases, different mechanisms that enhance the spherocytosis of cells and ApoBDs by macrophages, neutrophils, and other IS cells are beginning to be explored, although many more studies are still required in this field [143,144,145].

A final strategy consists of therapy directed specifically to the contents of the EVs. For this, it is essential to characterize the interior of the EVs to develop therapeutic strategies that precisely inhibit some of these components. A characteristic example would be to specifically target the microRNAs contained in the extracellular vesicles in such a way that they are silenced, or their expression is decreased to modulate the inflammatory response [146,147].

On the whole, despite the potential to specifically inhibit cell trafficking, especially of EVs, more studies are required to evaluate their therapeutic efficacy in IMIDs.

#### 3.2.2. Therapeutic Use of Extracellular Vesicles

Although EVs and the endomembrane system may represent a therapeutic target of interest, strategies are also being developed that are aimed at modifying and using EVs to treat different pathologies, such as IMIDs. Currently, EVs that are being investigated for therapeutic purposes can be natural (derived from cells) or can be modified through bioengineering and biotechnological processes [148]. The most commonly used EVs are those derived from mesenchymal stem cells (MSCs). This type of EV has shown an important anti-inflammatory and regenerative power, with a good safety profile, low immunogenicity, and a great capacity to cross biological barriers. They still have some important limitations, especially in their methods of production, characterization, quantification, pharmacokinetics, targeting, and transfer to target tissues [149]. Some studies have demonstrated the impact of EVs derived from MSCs in the prevention and treatment of autoimmune and inflammatory diseases in animal models [150,151]. One of the most important mechanisms by which these vesicles show an immunomodulatory effect lies in their ability to act on macrophages, decreasing their polarization towards proinflammatory M1 macrophages and increasing the M2 anti-inflammatory population [152]. Likewise, the use of EVs derived from MSCs in experimental models of asthma leads to overexpression of IL-10 and TGF-β1, which in turn promotes a decrease in Th2/Th1 populations, decreases the number of eosinophils, and increases the population of regulatory T lymphocytes as well as hypersensitivity and pulmonary inflammation [153]. Different EVs from various types of parasites, anti-inflammatory M2 macrophages, and even autologous transfer have been used to effectively reduce the inflammation that occurs in animal models of inflammatory bowel disease, although many more studies are still required in this area [130].

On the other hand, EVs can be functionalized through surface engineering to provide vesicles with greater target specificity and develop personalized therapeutic applications for inflammatory diseases. These strategies can be directed to the cell surface before the release of the EVs (by protein fusion or genetic engineering) or by chemical modifications of the EVs. Some of the molecules used to modulate the IS include different groups of peptides, small molecules, and antibodies [17]. In this context, one of the challenges faced by the use of EVs in the treatment of different inflammatory pathologies is the rapid elimination of them by the IS. Thus, through the development of functionalized EVs, elimination by immune cells could be avoided, and different components that specifically target a given tissue could be simultaneously added [18]. Some of the most important components in EVs that can increase their bioavailability are polyethylene glycol (increases the circulation time) and CD47 (inhibits uptake and clearance from the circulation by macrophages), whereas the presence of PS reduces their bioavailability [148]. Simultaneously, components found naturally in EVs such as tetraspanins, integrins, and lipids, or added through engineering processes, such as LAMP2, proteins fused with lactadherin, peptides bound to GPI, and magnetic particles conjugated with transferrin bound to its receptor in the EV, can significantly modulate the specificity towards the target organ [148].

In the same way, EVs can be used as carriers of different therapeutic agents, whether pharmacological or biological. In this sense, two main strategies can be used: either the cells are treated with the agent that is to be loaded before they produce and release the EVs, or it is conducted by different methods after being produced (incubation, electroporation, sonication, extrusion, etc.) [154]. Such EVs have shown efficacy as nanocarriers of different anti-inflammatory agents and mediators of the resolution of the immune response [155]. Zhuang et al. [156] developed three animal models of brain inflammation: (1) induced by lipopolysaccharides, (2) experimental autoimmune encephalitis, and (3) based on the GL26 brain tumour model. They evaluated the efficacy of loading EVs with either curcumin or STAT-3 inhibitors against inflammation. Importantly, they found benefits after the intranasal administration of these loaded vesicles in all three animal models, showing the potential worth of this strategy to reduce inflammatory processes in the brain.

The efficacy of the EV carrier strategy is also being evaluated in some IMIDs, such as rheumatoid arthritis, with some promising preliminary results [157]. Chen et al. [158] reported how the transfection of MSCs with a plasmid containing the microRNA miR-150-5p and the subsequent release of EVs with this microRNA reduced the inflammatory response in vivo, decreasing the expression of the metalloprotease MMP14 and vascular endothelial growth factor.

This type of approach has shown benefits in animal models of multiple sclerosis. Casella et al. [159] performed, by bioengineering processes, EVs derived from microglia that overexpress the lactadherin molecule, recognized by macrophages as an ‘eat me’ signal and loaded inside by the anti-inflammatory cytokine IL-4. Their results showed a significant decrease in the inflammatory response and a reduction in tissue damage in vivo.

Other recent studies evaluating the potential use of EVs as carriers of different anti-inflammatory components are detailed by Hwang et al. [17].

## 4. Conclusions

Vesicular trafficking is a key process of intercellular communication in physiological and pathological conditions, mainly represented by the endomembranous system and the EV. These systems are especially important in IS cells, which capture and integrate information from the outside and simultaneously marshal an adequate response, such as by releasing different products according to the information received. Different therapeutic strategies targeting the endomembrane system and EVs during IMIDs have shown potential application value. Most of the studies are preclinical, showing some evidence in in vitro and in vivo models. This is due in large part to the complexity of these biological processes and the methodological and technical difficulties of developing appropriate therapies for each type of disease. Given the importance of the endomembrane system and EVs in the pathological functioning of the IS in IMIDs, we encourage researchers and clinicians to make efforts at translational studies in this novel field, in conjunction with more basic research that will help to uncover the different characteristics and functions of vesicular trafficking, as well as its biological implications.

## Figures and Tables

**Table 1:** Summary of the main proteins and molecules implicated in the biosynthesis of EVs.

Main Components Involved in the Biogenesis and Release of EVs	Exosomes	Microvesicles	Apoptotic Bodies	References
ESCRT complex (ESCRT 0-III)	ESCRT-0 and -I recruit cargos and ESCRT-II. ESCRT-II then recruits ESCRT-III and in turn, ESCRT-III promotes ILV budding.	ARRDC1 and other arrestins interact with HECT ubiquitin ligases (WWP1, WWP2, and Itch), ALIX, and Tsg101, thereby influencing ESCRT activation.	-	[41,42,47,49,82]
Vps4	Vps4 ensures final membrane scission in late endosomes and/or ESCRT recycling.	Vps4 is needed for MVs release.	-	[48,84]
Tsg101	Tsg101 is a subunit of the ESCRT-I complex, being involved in the ESCRT-dependent biogenesis of exosomes.	ARRDC1 and other arrestins interact with HECT ubiquitin ligases (WWP1, WWP2, and Itch), ALIX, and Tsg101, thereby influencing ESCRT activation.	-	[47,49,83]
ALIX	ALIX interacts with specific proteins of the ESCRT complex such as Tsg101—a subunit of ESCRT-I—and with syntenin, the adaptor protein of the proteoglycan syndecan.	ARRDC1 and other arrestins interact with HECT ubiquitin ligases (WWP1, WWP2, and Itch), ALIX and Tsg101, thereby influencing ESCRT activation.	-	[45,46,50,82]
Tetraspanins	Tetraspanins (CD9, CD63, CD81…) are important in the recycling pathways between plasma membrane and cellular organelles and regulate biosynthetic maturation and trafficking of exosomes. The inhibition of these tetraspanins lead to a decrease in exosome production and release. Another tetraspanin (CD6) directs MVB cargoes for lysosomal degradation.	Tetraspanins can also appear on the surface of plasma membrane and thus they can take part in MVs as well (specially CD9).	-	[51,52,87]
Rab family proteins	Rab proteins influence the biogenesis and content of exosomes: Rab31 promotes EGFR cargo in MVB; Rab27a/b regulates fusion of MVBs at the plasma membrane for exosome release; and Rab7 promotes the fussion of MVBs with lysosomes, although it is regulated by Rab31.	Rab22a seems to be involved in the biogenesis of MV under hypoxic conditions.	-	[35,60,85]
Lipids (ceramides and cholesterol)	Ceramide and cholesterol lipid domains recruit factors, including flotillins and the autophagy-related protein LC3.	The modification of the plasma membrane asymmetry in its lipid contents can be implicated in the production of MVs through different mechanisms including aminophospholipid translocases, translocation on the outer plasma membrane of the acid sphingomyelinase, and by modification of the lateral pressure of phospholipids via phosphatidylserine (PS)-binding protein on the inner leaflet or sphingomyelin/cholesterol-binding protein on the outer leaflet.	-	[61,64,65,87]
Arrestins	Arrdc1 is implicated in the biogenesis of exosomes, although the mechanism is not fully understood.	ARRDC1 and other arrestins interact with HECT ubiquitin ligases (WWP1, WWP2, and Itch), ALIX, and Tsg101, thereby influencing ESCRT activation.	-	[46,82,83]
ADP ribosylation factors	ARF6 and PLD2 influence syntenin-ALIX ILV formation and subsequent exosomal release.	ARF6, PLD2, and ARF1 also induce the biosynthesis of microvesicles via activation of RhoA.	-	[45,80]
SNAREs	SNAREs mediate the fussion of MVBs with the plasma membrane, thus permitting exosome release.		-	[36,57,58,59]
Rho family and effectors	-	RhoA induces the phosphorylation of the myosin light chain (MLC) via a Rho-associated protein kinase (ROCK) signalling pathway.	ROCK signalling pathway is a pivotal regulator of the first step of apoptosis (membrane blebbing), therefore influencing later formation of ApoBDs.	[80,97]
Phosphatidylserine	-	-	A critical and exclusive marker of ApoBDs. PS is a phospholipid that is normally found in the inner membrane, but after apoptosis initiation it is translocated to the outer membrane, acting as a signal for its phagocytosis.	[94]
PANX1	-	-	PANX1 is a negative regulator of apoptopodia and beaded apoptopodia formation.	[99,100]
PlexB2	-	-	PlexB2 is implicated in the formation of apoptopodia and beaded apoptopodia.	[100]
